# Effect of drug-coated balloons in treatment of stenosis of the femoral artery and vein bypass graft not responding to plain old balloon angioplasty: a case report

**DOI:** 10.1186/s40792-019-0764-9

**Published:** 2019-12-23

**Authors:** Sohei Matsuura, Kota Yamamoto, Takafumi Akai, Toshihiko Isaji, Toshio Takayama, Katsuyuki Hoshina

**Affiliations:** Division of Vascular Surgery, Department of Surgery, The University of Tokyo, 7-3-1, Hongo, Bunkyo-ku, Tokyo, 113-8655 Japan

**Keywords:** Drug-coated balloon, Vein bypass graft, Restenosis, Plain old balloon angioplasty, Intimal hyperplasia

## Abstract

**Background:**

The use of drug-coated balloons (DCBs) with anti-proliferative agents in treating femoropopliteal lesions was approved in Japan in 2017. A better limb salvage rate or amputation-free rate of DCBs relative to plain old balloon angioplasty (POBA) has been reported; however, there is little evidence of the direct effect on intimal hyperplasia (IH).

**Case presentation:**

A 70-year-old man with chronic limb-threatening ischemia and foot gangrene had undergone bypass surgery from the left common femoral artery to the dorsalis pedis artery 2 years earlier. We evaluated the bypass graft using ultrasonography and found stenosis around the proximal anastomotic site, presumably due to IH. POBA was performed every 3 months due to the repeated re-stenosis of the lesion. Since using the DCB, no restenosis has been detected to date (10 months).

**Conclusion:**

DCB might be an effective tool for treating re-stenosis due to IH or vein grafts that do not respond to POBA.

## Introduction

Despite the remarkable advances in endovascular therapy, autogenous vein graft for peripheral arterial bypass remains the first choice for long occlusions or below-knee lesions due to its excellent patency [1, 2]. However, considering that approximately 30% of bypasses were reported to occlude within the first year after bypass surgery [3, 4], follow-up is critical for improving patency. In our department, we survey bypasses using duplex ultrasonography (DUS), which is noninvasive and low cost, every 3 months. We evaluate stenosis based on the elevation of the peak systolic velocity (PSV) of the artery or vein graft and consider a value of more than approximately 500 cm/s indication for re-intervention.

We have typically used plain old balloon angioplasty (POBA) for this re-intervention; however, we have sometimes encountered patients that were nonresponsive to POBA. The drug-coated balloon (DCB) has recently emerged and is expected to be a powerful tool in replacing POBA as it can achieve excellent outcomes, including increased limb salvage rate or amputation-free rate in the treatment of femoropopliteal lesions, which has recently been reported. However, there are only a few reports suggesting that DCB is beneficial for bypasses using autogenous grafts [5–8]. Among the various methods of improving the patency, the effect of anti-proliferative drugs on intimal hyperplasia (IH) of the anastomotic site is particularly attractive.

In this study, we report the case of a patient with chronic limb-threatening ischemia (CLTI) who underwent distal bypass, in which DCB was used in the treatment of repeated stenosis around the anastomotic site, presumably due to IH, and demonstrated the effect of the DCB.

## Case presentation

**Fig. 1:**
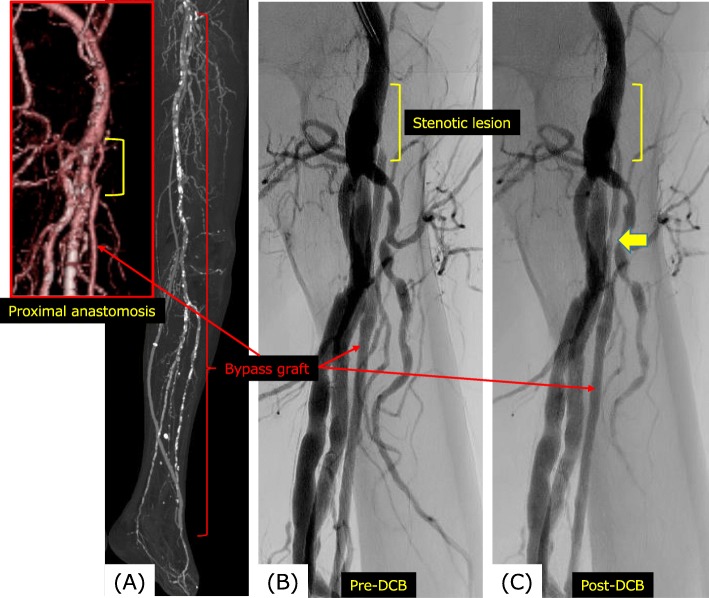
**a** Computed tomography angiography after distal bypass surgery. The reversed saphenous vein bypass is shown with a red bracket. The proximal anastomotic site was focused in another window. **b** Intra-arterial digital angiography before percutaneous transluminal arterioplasty with drug-coated balloons (DCBs). The stenosed area of the reversed saphenous vein graft is shown with a yellow bracket and the bypass graft is shown with a red arrow. **c** Intra-arterial digital angiography after PTA with DCB. The stenotic lesion was successfully dilated using a DCB. Another re-stenotic lesion was shown below without PSV increase, for which we dilated with DCB

A 70-year-old man with CLTI and foot gangrene underwent the bypass from the left common femoral artery to the dorsalis pedis artery using a reversed saphenous vein graft 2 years earlier (Fig. 1a). During DUS surveillance, we initially found severe stenosis around the femoral artery, anastomotic site, and vein bypass graft, where the PSV was increased to 564 cm/s, and consequently planned re-intervention (Fig. 1b). His medical history included multiple coronary risk factors, including hypertension, diabetes mellitus, and associated nephropathy requiring hemodialysis, unstable angina, left thalamic hemorrhage, and ex-smoker status. Above all, his cardiac function had drastically deteriorated after repeated coronary intervention performed for the unstable angina. The ejection fraction finally decreased to below 20%, which made it impossible to perform open surgery under general anesthesia. We had no choice but to select endovascular intervention (POBA) under local anesthesia.

### POBA

We performed a percutaneous puncture of the contralateral common femoral artery and POBA under the guidance of a 0.014-in. micro guide wire and 4.5-Fr guiding sheath. The first time, we used a 1.5 × 20-mm Coyote Balloon Dilation Catheter (Boston Scientific, Natick, MA, USA) and 2.0 × 40-mm and 3.0 × 60-mm SABER PTA dilatation catheters (Cordis, Santa Clara, CA, USA). The second time after 111 days later, we used 2.0 × 40-mm and 3.0 × 40-mm SABER PTA dilatation catheters. The third time after 115 days, we used 2.0 × 20-mm and 3.0 × 20-mm Rapidstream balloon catheters (Nipro Corporation, Osaka, Japan). In the final treatment using POBA after another 67 days, we used a 2.5 × 40-mm SABER PTA dilatation catheter and a 3.0 × 100-mm Rapidstream balloon catheter. All POBA was performed under nominal recommended pressure (8 atm for 3 mm). Dilatation was temporarily achieved after each procedure.

### DCB

We used a 4.0 × 60-mm IN.PACT Admiral DCB (Medtronic Vascular, Santa Clara, CA, USA), whose excipient paclitaxel concentration was 3.5 μg/mm^2^, after pre-dilatation using 2.0 × 60-mm and 3.0 × 60-mm Rapidstream balloon catheters (Fig. 1c). Because the 4.0 mm in diameter was oversized, we dilated the balloon to less than the nominal recommended pressure (8 atm), which approximated 2 or 3 atm. The range of the DCB covered the site which appeared slightly stenotic (Fig. 1c arrow) without increase of PSV via DUS.

### Change of PSV

**Fig. 2:**
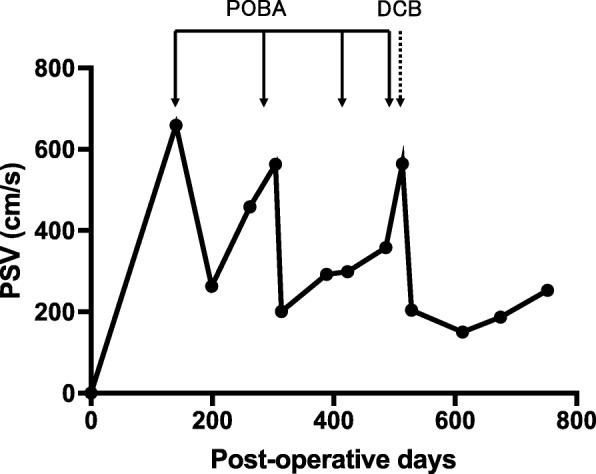
Change in the peak systolic velocity (PSV) of the vein graft postoperatively. The solid arrows show angioplasty with a plain balloon and the broken arrow shows angioplasty with a drug-coated balloon. The change in PSV of the vein graft after surgery is shown on the graph

We had set the threshold for the PSV value at 500 cm/s for re-intervention after bypass surgery and regularly monitored the PSV in this patient. During the follow up period, PSV increased beyond 500 cm/s twice (659 and 563 cm/s, respectively) and we performed POBA both times. The third and fourth POBA procedures were performed on the stenosis with PSVs of 299 and 358 cm/s because the bypass pulsation was found to be weakened remarkably. Thereafter, we performed DCB on the stenosis, the fifth re-intervention, when the PSV reached 564 cm/s. After the procedure using DCB was performed 10 months earlier, no remarkable graft stenosis has been noted till date. (Fig. 2).

## Discussion

DCB with anti-proliferation agents, such as paclitaxel or everolimus, was approved for femoropopliteal lesions in Japan in 2017 according to evidence from some randomized trials that demonstrated the superiority of DCBs over POBA in treatment [9–16]. However, the effect of DCBs on vein bypass grafts is controversial; Bjorkman et al. showed a trend in favor of DCBs in bypass grafts [5], but other reports did not demonstrate the superiority of DCBs over POBA [6–8]. As there are various causes of graft failure, these studies might lack the patient cohort volume to reveal a drug effect on IH. In our report, we focused on localized stenosis, presumably due to IH, and performed angioplasty on the same targeted lesion, which might make it possible to reveal the DCB effect more clearly.

Paclitaxel has multifunctional effects on IH and restenosis by inhibiting (1) proliferation of smooth muscle cells and fibroblasts, (2) migration of these cells, and (3) secretion of the extracellular matrix. Paclitaxel diffuses through the arterial wall to maintain high concentrations in the deeper smooth cells and fibroblast layers; however, it experiences quick wash-out from the luminal surface, which enables healing of endothelial cells [17]. These features of paclitaxel might cause a high anti-proliferating effect on IH and restenosis.

One limitation of this report was the difference in balloon diameter between the POBA and DCB. We had no choice but to select an oversized DCB due to a lack of device variation. It is therefore possible that our outcome comparison between DCB and POBA is biased relative to instructions for use.

The systematic review and meta-analysis reported by Katsanos et al. revealed that the risk of death was significantly increased after the first year following application of paclitaxel-coated balloons and stents in the femoropopliteal artery of the leg in patients with intermittent claudication [18]. It gave rise to heated discussion and an FDA statement required informed consent regarding the possibility of increased risk of long-term mortality when using paclitaxel-coated balloons and paclitaxel-eluting stents. In addition, the FDA warned that alternative treatment options for peripheral arterial disease should be discussed. However, they also stated that “for some individual patients at particularly high risk for restenosis, clinicians may determine that the benefits of using a paclitaxel-coated product may outweigh the risks [19].”

In our case, we explained the risks of long-term mortality according to the FDA statement and data from Katsanos et al.’s report and obtained informed consent. The patient expected the graft salvage not to lessen the activities of daily living. He found the need for repeated intervention during a short period of time tiring. We recognize that the merits and demerits of DCB offset each other. We assume that DCB for a localized lesion, theoretically associated with IH, may be acceptable.

## Conclusion

We presented the case of a patient who underwent distal bypass and developed repeated stenosis over a short period around the anastomotic site, for which treatment with DCB was effective. DCB might be an effective tool in treating re-stenosis due to IH or vein grafts that do not respond to POBA.

## Data Availability

All data generated or analyzed during this study are included in this published article.

## Abbreviations

CLTI: Chronic limb-threatening ischemia
DCB: Drug-coated balloon
DUS: Duplex ultrasonography
IH: Intimal hyperplasia
POBA: Plain old balloon angioplasty
PSV: Peak systolic velocity
